# HPV-based cervical cancer screening in Nicaragua: from testing to treatment

**DOI:** 10.1186/s12889-020-08601-z

**Published:** 2020-04-15

**Authors:** Francesca Holme, Francisco Maldonado, Orlando B. Martinez-Granera, Jose Maria Rodriguez, Juan Almendarez, Rose Slavkovsky, Pooja Bansil, Kerry A. Thomson, Jose Jeronimo, Silvia de Sanjose

**Affiliations:** 1 PATH, Department of Sexual & Reproductive Health, 2201 Westlake Ave., Suite 200, Seattle, WA 98101 USA; 2 Movicancer, Rpto. Las Palmas, del Semáforo “El Guanacaste” (Walmart), 200 mts. al Lago, 175 mts. al Este., Casa, #1108 Managua, Nicaragua; 3 Jose Jeronimo Consulting, Damascus, MD 20872 USA

**Keywords:** Cervical cancer, Screening, HPV test, Self-sampling, Follow-up, Treatment, Nicaragua

## Abstract

**Background:**

In Nicaragua, cervical cancer is the leading cause of cancer death among women. Human papillomavirus (HPV) testing, primarily using self-sampling, was introduced between 2014 and 2018 in three provinces. We analyzed data from the HPV screening program with the goal of describing key characteristics including reach, HPV prevalence, triage and treatment, and factors associated with follow-up completion.

**Methods:**

We analyzed individual-level data from routinely collected forms for women attending HPV-based cervical cancer screening. HPV-positive women were triaged with Pap or visual inspection with acetic acid (VIA) prior to treatment. Logistic regression was used to identify factors associated with receiving triage and treatment; analyses were adjusted for province, age, and self- vs. provider-collected sampling.

**Results:**

Forty-four thousand six hundred thirty-five women were screened with HPV testing; 96.6% of women used self-sampling. Six thousand seven hundred seventy-six women were HPV positive (15.2%), 54.0% of screen-positive women received triage, and 53.1% of triage-positive women were treated, primarily with cryotherapy. If women lost at triage are included, the overall treatment percentage was 27.8%. Province and provider sampling were significantly associated with completing triage. Province and triage type were significantly associated with receiving treatment. The odds of receiving treatment after Pap triage as compared to VIA was significantly lower (aOR: 0.05, 95% CI: 0.04–0.08, *p* < 0.001), and the relative proportion of women receiving treatment after Pap triage versus VIA was 0.29.

**Conclusions:**

Introduction of HPV testing resulted in a substantial number of women screened, and acceptance of self-sampling was high. Management of screen-positive women remained a challenge, particularly with Pap triage. Our results can inform other developing countries as they work to reach World Health Organization (WHO) elimination targets.

## Background

Although excellent tools exist today to prevent cervical cancer, the disease still kills over 300,000 women annually, with over 90% of these deaths occurring in developing countries [1]. While the Latin American region has experienced economic growth over the past several decades, pockets of extreme poverty and social disadvantage remain, and these communities are disproportionately affected by cervical cancer. In Nicaragua, cervical cancer is the leading cause of cancer death among women [1]. Over half of the country’s incident cases are detected by age 49 [2], endangering the health and economic well-being of individuals, families, and communities when women are in the prime of their adult lives. The Nicaraguan Ministry of Health’s (MINSA) efforts to control cervical cancer have included a nationwide program offering Pap testing in each health region and a new national cytology laboratory in Managua as of 2014. However, the impact of Pap testing is inherently limited by characteristics such as low sensitivity, the need to rescreen women every three years [3] and dependence on a large cytology infrastructure.

Human papillomavirus (HPV) testing is a superior screening method compared to Pap, offering higher sensitivity and a better long-term predictive value [4, 5]. A major advantage is that viral detection can be performed in self-collected vaginal cells, thus avoiding a speculum exam while producing more objective results than Pap. HPV tests can be performed in a local or regional laboratory by a trained technician, facilitating faster reporting of results. When combined with an adequate system for following up screen-positive women and access to efficient treatment, HPV testing has the potential to reduce cervical cancer incidence and mortality in low-resource settings, overcoming some of the challenges associated with previous screening methods [6–9]. The demonstrated clinical utility of HPV testing along with availability of highly effective HPV vaccines has contributed to a global landscape in which the World Health Organization (WHO) has called for the elimination of cervical cancer. Countries are called upon to set programmatic goals that include, among others, screening of 70% of women twice between the ages 35 to 45 and treatment of 90% of screen-positive women [10].

From 2014 to 2018, the global health organization PATH worked closely with MINSA in Nicaragua through the local NGO Fundación Movicancer to introduce HPV testing using QIAGEN’s *care*HPV™ assay within the public sector health system in three provinces as part of the “Scale-Up” project. “Scale-Up” was a regional effort to introduce and scale up HPV testing for cervical cancer screening in four Central American countries. We analyzed HPV screening program performance in Nicaragua in 2017 within the public sector health system, when screening activities under the Scale-Up project were fully underway. Our goal was to describe key characteristics of the program including reach, HPV prevalence, triage and treatment, and factors associated with follow-up completion. These results, including successes and challenges encountered in program delivery, can inform other low- and middle-income countries (LMICs) as they work to reach WHO elimination goals.

## Methods

**Fig. 1:**
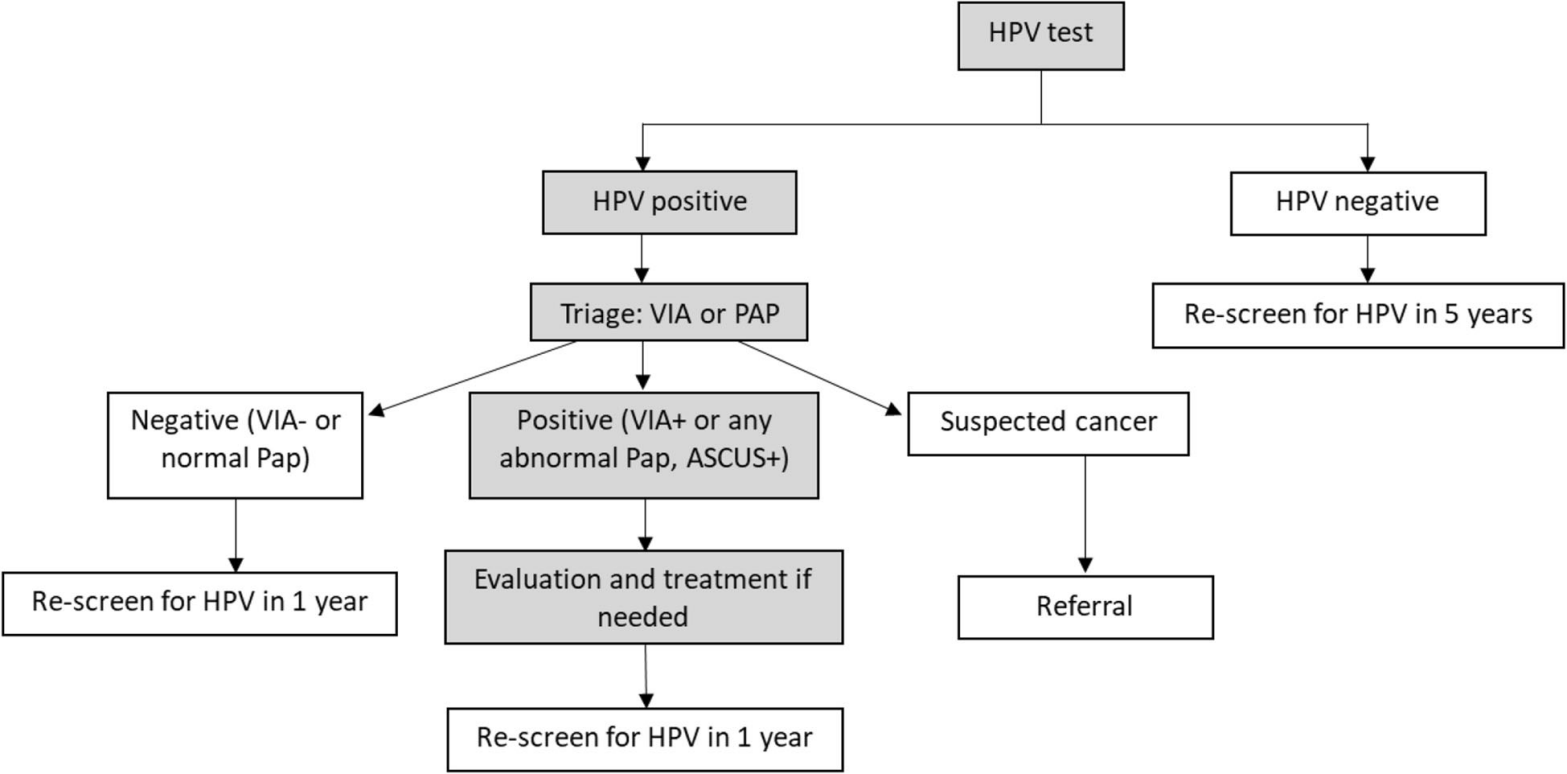
Overview of HPV-based screening, triage, and treatment algorithm in Nicaragua. HPV: Human papillomavirus. VIA: Visual inspection with acetic acid. Shaded areas represent the steps under evaluation in this report

Details of the overall Scale-Up project methods have been described elsewhere [11]. This report is a secondary analysis, using data from the public sector health system in Nicaragua. MINSA defined the target population for screening with HPV tests as all women aged 30 to 59 years living in the provinces of Chinandega, Chontales, and Carazo (141,637 women) [12]. These provinces are generally representative of most of Nicaragua’s geographic territories and populations: Carazo is closest to Managua and is the most urbanized, densely populated and wealthiest of the three; Chontales is primarily rural with a disperse population and is more economically depressed; and Chinandega contains both large urban centers and significant rural territory with populations living in a wide range of economic circumstances. Absent are communities and conditions typically found on the more ethnically diverse and remote provinces of the Atlantic coast of Nicaragua. Women were screened opportunistically in public clinics and during community outreach campaigns. Women were first encouraged to collect their own vaginal samples and were offered a simple brush and tube along with printed visual instructions and verbal coaching by a nursing assistant, nurse, or community health worker. If a woman preferred a clinician-collected sample, she could have one upon request. Local management algorithms specified that women who screened negative for HPV would be re-screened in 5 years, while screen-positive women were referred for additional care. The subsequent care included a “triage” step, wherein women underwent either Pap or visual inspection with acetic acid (VIA) (Fig. 1) depending on the capacity of the clinic. Women with any abnormal Pap result (atypical squamous cells of undetermined significance [ASCUS] or greater) were referred to colposcopy followed by treatment of any visible lesion (biopsy confirmation of disease was not required by the Nicaraguan management algorithm for HPV positive women, even in suspected HSIL cases). Women with a VIA-positive evaluation were treated immediately or referred for further care if needed, and women with a VIA-negative evaluation were advised to return in 1 year for rescreening. In 2017, MINSA offered cryotherapy and excision treatment for precancer cases, as well as hysterectomy, radiation, and chemotherapy in cases of cancer.

The 2017 data of interest for this analysis were extracted from forms and logs routinely collected in the public sector health system on cervical cancer screening visits and follow-up. De-identified data, including province, age, sampling method (self- or provider-collected), HPV test result, triage visit with Pap or VIA, triage result, treatment visit, and treatment method were sent to PATH for analysis. We conducted descriptive analyses and generated 95% confidence intervals (CI) where relevant. Time elapsed between screening, triage, and treatment among those managed with Pap versus VIA was assessed and compared using a T-test. We used both crude and adjusted logistic regression models to identify whether province, age, screening modality, or triage method was associated with receiving triage and treatment among screen-positive women; results are reported as odds ratios (OR) and 95% confidence intervals (95% CI) as compared to not receiving triage or treatment. All statistical analyses were conducted in Stata 13.1 (Statacorp, College Station, TX). This secondary analysis of de-identified data was determined not to be human subjects research by PATH’s research determination committee and thus was exempt from ethics committee review.

## Results

### Screening program characteristics and HPV positivity

A total of 44,635 women were screened with HPV tests across the three provinces in 2017 (Table 1). The largest proportion of women screened (47.8%) was in the Chinandega province. The mean age of women across all three provinces was 40.8 years (standard error [SE]: 8.6) and nearly all women (99.1%) were within the target age range of 30 to 59 years. Nearly all women (96.6%) collected their own vaginal samples.

**Table 1:** HPV screening activity and test results by sociodemographic and programmatic characteristics among women screened for cervical cancer in Nicaragua in 2017

	Total		HPV Positive	
	N	%	N	Prevalence % (95% CI)
**Total**	44,635	100	6776	15.2 (14.9–15.5)
**Department**				
Carazo	12,261	27.5	1962	16.0 (15.4–16.7)
Chinandega	21,333	47.8	3028	14.2 (13.7–14.7)
Chontales	11,041	24.7	1786	16.2 (15.5–16.9)
**Age, years**				
**Mean (SE)**	40.8 (8.6)	N/A	39.5 (8.5)	N/A
**Median (IQR)**	39.0 (33–47)	N/A	37.0 (20–71)	N/A
**In target age range (30–59 years)**	44,222	99.1	6681	98.6
**Out of target age range**	376	0.8	91	1.3
< 25	39	0.09	9	23.1 (12.4–39.0)
25–29	183	0.4	47	25.7 (19.9–32.5)
30–34	13,255	29.7	2456	18.5 (17.9–19.2)
35–39	9485	21.3	1459	15.4 (14.7–16.1)
40–44	7200	16.1	1011	14.0 (13.3–14.9)
45–49	5744	12.9	715	12.5 (11.6–13.3)
50–54	4700	10.5	562	12.0 (11.1–12.9)
55–59	3838	8.6	478	12.5 (11.4–13.5)
60+	154	0.3	35	22.7 (16.8–30.0)
Missing	37	0.08	4	10.8 (4.1–25.8)
**HPV Screening Method**				
Self-collected	43,105	96.6	6578	15.3 (14.9–15.6)
Provider-collected^a^	637	1.4	112	17.6 (14.8–20.7)
Unknown	893	2.0	86	9.6 (7.9–11.7)

*Abbreviations*: *HPV* Human papillomavirus, *SE* standard error, *IQR* Interquartile range, *CI* confidence interval

^a^133 women were screened by both modalities; these women have been included in the provider-collected category, as there was generally either a real or perceived problem with the self-collected sample that required a provider to recollect the sample, and this provider-collected sample was used for HPV testing

HPV positivity ranged among provinces from 14.2% (95% CI 13.7–14.7) in Chinandega to 16.2% (95% CI 15.5–16.9) in Chontales, with a value of 16.0% (95% CI 15.4–16.7) in Carazo. Chinandega had significantly lower HPV positivity compared to the other two provinces. HPV prevalence varied by age group; some differences were statistically significant (Table 1). Women aged 25 to 29 years had the highest HPV prevalence of 25.7% (95% CI 19.9–32.5), followed by a significant drop in the 30 to 34 year age group to 18.5% (95% CI 17.9–19.2). Prevalence generally declined through subsequent age groups to 12.5% among women aged 55 to 59 years (95% CI 11.4–13.5) and then increased significantly to 22.7% in women aged 60 years or older (95% CI 16.8–30.0). HPV prevalence was slightly lower in women with self-collected samples (15.3, 95% CI 14.9–15.6) as compared to provider-collected samples (17.6, 95% CI 14.8–20.7), but this difference did not reach statistical significance.

### Triage of HPV positive women

Among the 6776 HPV positive women, 3656 (54.0%) had a triage test recorded (Table 2); the remaining 3120 (46.0%) were considered lost at the triage step. Overall, 2784 (76.1%) of the HPV-positive women who were triaged received Pap while the remaining 872 (23.9%) received VIA. The proportion of VIA triage varied by province, with Chontales using VIA for the highest proportion (60.9%) and Chinandega the least (15.9%) (data not shown).

**Table 2:** Triage by method, treatment and time elapsed in follow-up of HPV positive women in Nicaragua in 2017

	Total Triaged N (%)	Triaged with Pap N (%)	Triaged with VIA N (%)	***P*** value‡
**Women triaged**	3656 (100)	2784 (100)	872 (100)	< 0.001
**Triage result**				
Negative	2293 (62.7)	1851 (66.6)	442 (50.7)	< 0.001
Positive^a^	915 (25.0)	513 (21.7)	402 (46.1)	< 0.001
Unknown	448 (12.3)	420 (15.1)	28 (3.2)	< 0.001
**Treated**	486 (13.3)	132 (4.7)^b^	361 (41.4)	< 0.001
**Percent treated among triage-positive women**	53.1	25.7	89.8	
**Number of days from screening to triage**				
**N (women with available data)**	2566 (70.2)	2089	477	
**Mean (SD)**	61.9 (SD: 43.8)	58.1 (SD: 37.7)	78.6 (SD: 61.5)	< 0.001
**Number of days from triage to treatment**				
**N (women with available data)**	470 (51.3^c^)	116	318	
**Treated on same day**	263 (56.0)	2 (1.7)	262 (82.4)	< 0.01
**Mean days if not treated on same day (SD)**	89.2 (SD 86.5)	103.8 (SD 93.4)	75.2 (SD 83.6)	0.05

*Abbreviations*: *VIA* visual inspection with acetic acid, *SD* standard deviation

^a^A Pap result of atypical squamous cells of undetermined significance (ASCUS) or greater in the triage step was considered triage-positive and therefore required treatment according to the local management algorithm

^b^There were 82 cases classified as high-grade squamous intraepithelial (HSIL) in Pap triage, of which 25 were treated (30.5%)

^c^Percentage of triage-positive women with available data

‡*P*-value comparing HPV positive women triaged with Pap versus VIA

### Triage and treatment among women managed with Pap, VIA

Among the women triaged with Pap, 513 women (21.7%) received a result of ASCUS or greater (ASCUS+), and 15.1% did not have Pap results recorded in our available data (Table 2). Among women with a Pap result of ASCUS+, 117 had an ASCUS result (22.8%), 308 had a low-grade squamous intraepithelial lesion (LSIL) result (60.0%), 82 had a high-grade squamous intraepithelial lesion (HSIL) result (16.0%), and 6 had a result of cancer (1.2%) (data not shown); these results were not biopsy-confirmed in our available dataset. Among women triaged with Pap, 132 women were treated; this represents 25.7% of women with an ASCUS+ Pap result in triage. Considering only those women with an HSIL or cancer result (HSIL+) in Pap triage, 27 (30.7%) were treated; this percentage did not differ significantly from those women with an LSIL or ASCUS (LSIL-) result in Pap triage (*p* = 0.243; data not shown).

Among women triaged with VIA, 402 (46.1%) received a VIA positive result and 28 (3.2%) had no VIA result recorded (Table 2). Among women triaged with VIA, 361 were treated; this represents 89.9% of VIA-positive women in triage.

### Time elapsed between screening, triage and treatment steps

Among triaged women, 70.2% of women had a mean time elapsed between screening and triage recorded, and 51.3% of triage-positive women likewise had mean time elapsed between triage and treatment recorded. The mean time elapsed between HPV sample collection and Pap collection for triage was 58.1 days (standard deviation [SD]: 37.7); among women triaged with Pap for whom data were available on time between visits, 2 were treated on the same day as triage. For remaining women in this category, mean time from Pap triage to treatment was 103.8 days (SD: 93.4) (Table 2). Among HSIL+ women, the mean time elapsed between triage and treatment was 158.4 days (SD: 156.8). This was significantly longer than for those women with LSIL- of 83.6 days (SD: 63.9, *p* < 0.001; data not shown). The mean time elapsed between HPV sample collection and VIA triage exam was 78.6 days (SD: 61.5); for women triaged with VIA for whom data were available on time between visits, the majority (82.4%) were treated the same day. For remaining women in this category, mean time from VIA triage to treatment was 75.2 days (SD: 83.6 days), which was not statistically significantly different from women triaged with Pap. Data on time between HPV sample collection and delivery of test result were not available.

### Treatment modalities

Among those with a record of treatment, 472 women were treated with cryotherapy (97.1%) and 14 (2.9%) received advanced procedures including 5 hysterectomies, 7 cold knife conizations, 1 chemotherapy and 1 radiotherapy (data not shown). All excision treatments occurred in the Chontales province.

### Factors associated with triage, treatment completion

In adjusted analyses, both province and screening modality were significantly associated with a woman completing the triage step (Table 3). After adjusting for age and screening modality, the odds of a woman completing triage were 2.78 times higher for women residing in Chontales (95% CI: 2.43–3.19, *p* < 0.001) and 1.23 times higher for women residing in Chinandega (95% CI: 1.09–1.38, *p* < 0.001) compared to women residing in Carazo. After adjusting for age and province, the odds of completing triage were 2.82 times higher for women who had a provider-collected sample compared to women who collected their own sample for HPV testing (95% CI: 1.82–4.39, *p* < 0.001).

**Table 3:** Factors associated with receiving triage among HPV screen positive women in Nicaragua in 2017

	Not Triaged (***N*** = 3120)	Triaged (***N*** = 3656)			
	n (%)	n (%)	Unadjusted Prevalence OR^**a**^ (95% CI)	Adjusted Prevalence OR^**b**^ (95% CI)	Triage Ratio
**Province**					
Carazo	1062 (54.1)	900 (45.9)	Ref	Ref	Ref
Chinandega	1482 (48.9)	1546 (51.1)	1.23 (1.10–1.38)*	1.23 (1.09–1.38)*	1.11
Chontales	576 (32.3)	1210 (67.8)	2.48 (2.17–2.81)*	2.78 (2.43–3.19)*	1.48
**Age, years**					
< 30	28 (50.0)	28 (50.0)	Ref	Ref	Ref
30–39	1812 (46.3)	2103 (53.7)	1.16 (0.68–1.97)	1.18 (0.69–2.02)	1.07
40–49	747 (43.3)	979 (56.7)	1.31 (0.77–2.23)	1.36 (0.79–2.34)	1.13
50–59	512 (49.2)	528 (50.8)	1.03 (0.60–1.77)	1.07 (0.62–1.86)	1.02
60+	19 (54.3)	16 (45.7)	0.84 (0.36–1.96)	0.89 (0.38–2.11)	0.91
Unknown	2 (50.0)	2 (50.0)	1.00 (0.13–7.60)	0.63 (0.08–4.97)	1.00
**HPV Screening Modality**					
Self-collected	3019 (45.9)	3559 (54.1)	Ref	Ref	Ref
Provider-collected	27 (24.1)	85 (75.9)	2.67 (1.73–4.13)*	2.82 (1.82–4.39)*	1.40
Unknown	74 (91.9)	12 (14.0)	0.14 (0.07–0.25)*	0.07 (0.04–0.14)*	0.26

*Abbreviations*: *HPV* Human papilloma virus, *CI* confidence interval, *OR* odds ratio, *Ref* Reference category

* *P* values < 0.001

^a^Unadjusted prevalence odds ratio for the association between each risk factor and receiving triage (either Pap or VIA)

^b^Adjusted prevalence odds ratio for all the listed factors

In adjusted analyses, both province and triage method were significantly associated with a woman receiving treatment (Table 4). After adjusting for age and triage method, women residing in Chinandega were 66% less likely to receive treatment than women residing in Carazo (aOR: 0.34, 95% CI: 0.20–0.56, *p* < 0.001). After adjusting for age, province, and screening modality, the odds of receiving treatment after Pap triage as compared to VIA was significantly lower (aOR: 0.05, 95% CI: 0.04–0.08, *p* < 0.001), and the relative proportion of women receiving treatment after Pap triage versus VIA was 0.29. There were no statistically significant associations observed between age and receipt of triage or treatment, or between screening modality (self- versus provider-collected sampling) and receipt of treatment. There was also no statistically significant association between a HSIL+ Pap result in triage and receiving treatment (data not shown).

**Table 4:** Factors associated with receiving treatment among HPV positive, triage positive women in Nicaragua in 2017

	Not treated (***N*** = 429)	Treated (***N*** = 486)			
	n (%)	n (%)	Unadjusted Prevalence OR^**a**^ (95%CI)	Adjusted Prevalence OR^**b**^ (95%CI)	Treatment Ratio
**Province**					
Carazo	50 (29.2)	121 (70.7)	Ref	Ref	Ref
Chinandega	199 (70.1)	85 (29.9)	0.18 (0.12–0.27)*	0.34 (0.20–0.56)*	0.42
Chontales	180 (39.1)	280 (60.9)	0.64 (0.44–0.94)**	0.67 (0.42–1.07)	0.86
**Age, years**					
< 30	5 (55.6)	4 (44.4)	Ref	Ref	Ref
30–39	247 (44.3)	311 (55.7)	1.57 (0.42–5.92)	1.26 (0.21–7.77)	1.25
40–49	102 (46.4)	118 (53.6)	1.44 (0.38–5.52)	1.37 (0.22–8.59)	1.21
50–59	71 (57.3)	53 (42.7)	0.93 (0.24–3.64)	1.13 (0.18–7.27)	0.96
60+	4 (100.0)	0 (0)	NA	NA	NA
Unknown					
**HPV Screening Modality**					
Self-collected	420 (46.8)	477 (53.2)	Ref	Ref	Ref
Provider-collected	9 (52.9)	8 (47.1)	0.78 (0.30–2.04)	1.11 (0.37–3.32)	0.89
Unknown	0 (0)	1 (100.0)	NA	NA	1.88
**Triage Method**					
VIA	48 (11.9)	354 (88.1)	Ref	Ref	Ref
Pap	381 (74.3)	132 (25.7)	0.05 (0.03–0.07)*	0.05 (0.04–0.08)*	0.29

*Abbreviations*: *HPV* Human papillomavirus, *CI* confidence interval, *OR* odds ratio, *Ref* Reference category, *NA* not applicable, *VIA* visual inspection with acetic acid

*P values < 0.001

**P value = 0.022

^a^Unadjusted odds ratio for the association between each risk factor and treated

^b^Adjusted odds ratio for all the listed factors

## Discussion

It is widely acknowledged that having an effective screening and treatment program is a crucial step toward ultimately reducing cervical cancer burden [10]. Our analysis of data from the first systemic collection of indicators for cervical cancer screening and treatment showed that HPV tests in Nicaragua reached approximately 31.5% coverage of the target population of the three provinces analyzed in 2017. If replicated in subsequent years, this approach could reach 100% coverage within the 5-year screening interval specified in Nicaraguan guidelines for HPV testing [13]. In order to achieve this coverage, MINSA would likely need to undertake a population-based outreach strategy rather than the opportunistic approach used here, and our data do not allow us to speculate on HPV test uptake under such a strategy. Although MINSA had substantial existing Pap infrastructure before HPV test introduction, it is unclear how HPV test coverage may compare to previous efforts because these indicators were not routinely monitored. While HPV test positivity varied by province, it was within the expected range for this region, including mirroring the U-shaped curve in HPV prevalence by age seen in other studies, with higher prevalence among younger and older women and a decline in middle age [14, 15]. The high percentage of self-collected HPV samples indicates a wide acceptance of this screening modality when it is offered as a primary outreach strategy. These programmatic successes were largely made possible by MINSA’s broad network of provincial and local health facilities extending dedicated resources and personnel time.

Although these successes in screening outreach and test performance are notable, the low overall triage and treatment percentages are of concern. Only half of the triage-positive women had documented treatment, and if the same triage-positive rates are applied for women lost at triage, the overall treatment percentage is just 27.8%, substantially lower than the 90% goal set by WHO in its elimination targets [10]. This estimate relies on several assumptions, including that women were truly lost in the triage step rather than receiving treatment elsewhere. In Guatemala and Honduras, where similar HPV testing strategies were implemented under the Scale-Up project, the percentages of triage-positive women treated between 2015 and 2018 were 84.7 and 58.8%, respectively [11]; and estimated percentages of women treated, accounting for those lost in the triage step, were reduced to 71.2 and 30.2%, respectively. Findings from an HPV-based screening program in Argentina, where the screening algorithm uses Pap and colposcopy for triage, 85.2% of women with confirmed CIN2+ lesions were treated; this represents just 27.7% of women with an abnormal Pap in triage [16]. Although limited, some of these findings are similar to Nicaragua’s and suggest that the Nicaraguan experience may point to challenges common to low-resource settings that will require specific interventions to retain women in the screening and treatment cascade.

Our analysis suggests that MINSA’s decision to triage most HPV-positive women with Pap rather than VIA is an important factor contributing to low triage and treatment percentages. Although data on time elapsed in the screening and treatment process were incomplete, available data show that while it took slightly more time for women to access VIA compared to having a Pap smear collected, the subsequent time to treatment was dramatically shorter because most women triaged with VIA received treatment the same day, whereas most women triaged with Pap had to wait for their results and receive treatment in a different appointment months later. Over 15% of women triaged with Pap never had a result recorded, likely because of the challenges presented by distance of and delay in cytology processing services. Even women with HSIL+ Pap results did not appear to be prioritized within the system for rapid follow-up, perhaps due to limited availability of more advanced treatment such as LEEP, although it is difficult to draw firm conclusions about this subgroup of women in the absence of histological confirmation of disease. Notably, over three times as many women who were triage-positive with VIA received treatment compared to those who were triage-positive with Pap. This is likely due, at least in part, to the multiple visits required for a complete Pap follow-up compared to VIA.

Evidence suggests that triaging with either Pap or VIA effectively nullifies the gain in sensitivity of testing with HPV for primary screening [17]. Cost-effectiveness models suggest that a screen-and-treat approach, in which all HPV-positive women are treated and VIA is used only to determine treatment eligibility (rather than to triage women), would be the most effective and cost-effective strategy for Nicaragua [18] and other countries in the region [19]. Alternatively, emerging evidence indicates that machine learning algorithms, which are currently under evaluation, could dramatically improve the quality of the visual triage step using digital images of the cervix [20], enabling providers to better “see-and-treat” with less overtreatment than we currently observe in an “HPV test-and-treat” algorithm.

Although the Nicaraguan management algorithm mirrors WHO guidelines in calling for a one-year follow-up visit for women who were HPV positive but negative in the triage step, unfortunately our data set did not include this indicator. In another evaluation nested within the Scale-Up project, we analyzed one-year follow-up attendance among HPV positive, triage negative women in Honduras. In that context, just 3.6% of women returned spontaneously. Health care providers were able to recall an additional 71.3% of women using phone calls and other reminder contacts. 36% of women returning for a one-year follow up appointment remained HPV positive, underscoring the importance of this follow-up step [21]. In Central America and elsewhere, health authorities may wish to consider these findings when designing their screening and treatment algorithms and delivering their screening programs.

Another finding was that women with clinician-collected samples were more likely to receive triage (although sampling modality was not associated with receiving treatment); our data do not provide insight into why these women had clinician-collected samples. While broad use of self-sampling is likely to be the most practical way for LMICs to screen 70% of their target populations in order to reach WHO elimination goals, it is important to consider additional programmatic implications of this approach. It may be that women who self-sample require more tailored outreach to encourage attendance at follow-up compared to women with provider-collected samples.

Our results also raise concerns about treatment modality in Nicaragua. According to MINSA and project field workers, cryotherapy was widely available in the provinces where HPV testing took place and stockouts of cryotherapy gas were rare, which is often not the case in low-resource settings [22]. However, the lack of availability of advanced treatment modalities was clearly a challenge, particularly in Carazo and Chinandega where no women were reported to have received excision treatment although studies in other populations suggest that about 15% of women with precancerous lesions could benefit from it [23, 24]. Some women seek care in the private sector and records of their treatment may be lost to the public system.

Histological data and cancer registry data were not available for our evaluation; thus, it is beyond the scope of the current analysis to confirm diagnoses or estimate cure rates. Nevertheless, our data give us important insight into the state of treatment efforts in 2017. Other LMICs are also likely to face the challenge of limited capacity for treatment. Reports from other countries indicate that thermal ablation is an effective and practical alternative to cryotherapy that may enable countries to increase their ablative treatment capacity [25, 26]. WHO guidelines issued in 2019 support the use of thermal ablation in LMICs [27].

Of note, in 2018 MINSA invested time and resources in finding and following up women who screened positive for HPV in previous years, including 2017. This effort led to additional women in the target provinces receiving treatment, for a total of 67.1% of HPV-positive, triage-positive women receiving treatment from 2015 to 2018 [11], and suggests that there is potential for LMICs to address the problem of loss to follow-up within a longer timeframe, given sufficient resources and prioritization.

Our analysis was possible because of a concerted effort by MINSA to implement more robust data collection practices, enabling tracing of screen-positive women through triage and treatment, analysis of individual data, and periodic review of key consolidated indicators. Weakness in existing health information system (HIS) infrastructure is likely to present challenges in many LMICs as they work to eliminate cervical cancer. Examples of cervical cancer screening-related HIS improvements from Nicaragua [28], Argentina [29], and Malaysia [30] provide practical models for countries seeking to ensure that their HIS enables them to effectively monitor treatment completion and overall screening program performance.

## Conclusions

Our analysis of 2017 data from Nicaragua contributes to a growing body of evidence suggesting that LMICs can make progress toward implementing more effective cervical-cancer screening programs, and that self-sampling can help LMICs overcome infrastructure barriers to reach their target populations. In order to ensure that these programs meet their intended goal of reducing cervical cancer morbidity and mortality and ultimately achieve the WHO’s cervical cancer elimination targets, new strategies and renewed commitment to streamline and strengthen follow-up, management, and treatment of screen-positive women are needed.

## Data Availability

The datasets used and/or analyzed during the current study are available from the corresponding author on reasonable request.

## Abbreviations

aOR: Adjusted Odds Ratio
ASCUS: Atypical squamous cells of undetermined significance
CIN: Cervical intraepithelial neoplasia
HIS: Health information system
HPV: Human papillomavirus
HSIL: High grade squamous intraepithelial lesion
LMICS: Low- and middle-income countries
LSIL: Low grade squamous intraepithelial lesion
MINSA: Nicaragua Ministry of Health
VIA: Visual inspection with acetic acid
WHO: World Health Organization
